# Multi-objectives reinforcement federated learning blockchain enabled Internet of things and Fog-Cloud infrastructure for transport data

**DOI:** 10.1016/j.heliyon.2023.e21639

**Published:** 2023-11-02

**Authors:** Mazin Abed Mohammed, Abdullah Lakhan, Karrar Hameed Abdulkareem, Mohd Khanapi Abd Ghani, Haydar Abdulameer Marhoon, Jan Nedoma, Radek Martinek

**Affiliations:** a Department of Artificial Intelligence, College of Computer Science and Information Technology, University of Anbar, Anbar, 31001, Iraq; b Department of Cybersecurity and Computer Science, Dawood University of Engineering and Technology, Karachi City 74800, Sindh, Pakistan; c College of Agriculture, Al-Muthanna University, Samawah 66001, Iraq; d Department of Software Engineering, Faculty of Information and Communication Technology, Universiti Teknikal Malaysia Melaka (UTeM), Malaysia; e Information and Communication Technology Research Group, Scientific Research Center, Al-Ayen University, Thi-Qar, Iraq; f College of Computer Sciences and Information Technology, University of Kerbala, Karbala, Iraq; g Department of Telecommunications, VSB-Technical University of Ostrava, 70800 Ostrava, Czech Republic; h Department of Cybernetics and Biomedical Engineering, VSB-Technical University of Ostrava, 70800 Ostrava, Czech Republic

**Keywords:** Self-autonomous vehicle, Blockchain, MORFLB, Agents, Training, Cloud

## Abstract

For the past decade, there has been a significant increase in customer usage of public transport applications in smart cities. These applications rely on various services, such as communication and computation, provided by additional nodes within the smart city environment. However, these services are delivered by a diverse range of cloud computing-based servers that are widely spread and heterogeneous, leading to cybersecurity becoming a crucial challenge among these servers. Numerous machine-learning approaches have been proposed in the literature to address the cybersecurity challenges in heterogeneous transport applications within smart cities. However, the centralized security and scheduling strategies suggested so far have yet to produce optimal results for transport applications. This work aims to present a secure decentralized infrastructure for transporting data in fog cloud networks. This paper introduces Multi-Objectives Reinforcement Federated Learning Blockchain (MORFLB) for Transport Infrastructure. MORFLB aims to minimize processing and transfer delays while maximizing long-term rewards by identifying known and unknown attacks on remote sensing data in-vehicle applications. MORFLB incorporates multi-agent policies, proof-of-work hashing validation, and decentralized deep neural network training to achieve minimal processing and transfer delays. It comprises vehicle applications, decentralized fog, and cloud nodes based on blockchain reinforcement federated learning, which improves rewards through trial and error. The study formulates a combinatorial problem that minimizes and maximizes various factors for vehicle applications. The experimental results demonstrate that MORFLB effectively reduces processing and transfer delays while maximizing rewards compared to existing studies. It provides a promising solution to address the cybersecurity challenges in intelligent transport applications within smart cities. In conclusion, this paper presents MORFLB, a combination of different schemes that ensure the execution of transport data under their constraints and achieve optimal results with the suggested decentralized infrastructure based on blockchain technology.

## 1 Introduction

**Figure 1:**
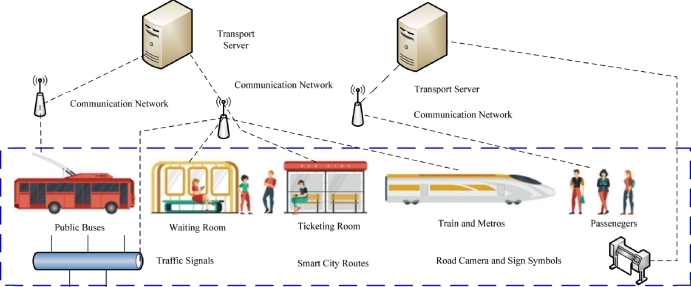
Public transport data scenario for end users.

These days, the integration of Internet of Things (IoT) devices for transport applications has been growing. For instance, mobile-based ticketing, route searching, public transport time-table, and trip planner applications are increasing [1] for various purposes. Smart city transportation encompasses public and private transportation executed inside the city with different routes. Intelligent transportation systems (ITS) level infrastructure enhances people's daily lives by providing data-driven decision-making support based on information collected from various IoT devices. ITS is implemented in buses, cars, trains, and other modes of public transportation, ensuring efficient transportation services inside smart cities. ITS combinations of different technologies, e.g., wireless 6G, software-defined networks, and cloud computing [2]. The end users' IoT devices, such as mobile devices, smartwatches, and sensors, offload data to the transport server for processing [3]. However, many service automation, security, and scheduling issues exist in the current ITS-enabled transport infrastructure for transport data, including user data privacy, service time, and cost for transport applications. We discuss the pictorial form transport data in a based scenario as illustrated in Fig. 1. In smart cities, public transport such as buses, metro, and trains are connected to the transport servers through communication networks. The transport data process on the transport servers along the camera, sign symbols, and signals. The passengers are also connected to the transport servers, where data can be accessed and offloaded data to the servers, as shown in Fig. 1.

These studies [1], [2] suggested a single objective level objective for the transport data and services in the secure form. The objective is to ensure data security at the ITS infrastructure level for transport application data. These studies [3], [4], [5], [6] suggested the decentralized security schemes and suggestions for the transport applications. The blockchain technologies with the private and public frameworks presented for ITS infrastructure with the cloud computing for transport applications. The machine learning and deep learning methods are integrated to optimize the transport's security in distributed transport networks. These studies [7], [8], [9], [10] suggested blockchain-enabled transport data sharing with the time and security multi-objectives. These studies minimized the security risk and communication time among public transport in ITS infrastructure networks. All these studies trained their machine learning models to identify the attacks based on trained samples in ITS infrastructure. Even though these studies showed that blockchain technologies are optimal for handling Phishing, malware, ransomware, denial of services, man in the middle, and SQL injections. However, many issues exist in the current blockchain based on ITS infrastructure when we optimize multi-objectives for transport data application in networks.

### 1.1 Research gap analysis and research questions

In our last publications [11], [12], [13], we optimized the multi-objectives of transport applications such as fault-tolerant, energy, latency, security, and resource consumption in blockchain-based transport systems. In this paper, we consider the following research questions (RQ) that have yet to be studied by state-of-the-art studies. However, including the aforementioned state-of-the-art studies, many research questions and gaps are available for further research. (i) All existing suggested machine learning and deep learning methods are only optimized for the entire process. However, processing data in blockchain after attacks on a single node takes an entire process, which is time and resource-consuming. (ii) All the existing blockchain-enabled security methods performed well on known attacks and controlled invalid transactions. However, unknown and untrained attacks consume much more resource time and incur higher delays, where transactions miss their deadlines. (iii) Therefore, the multi-objective problem in blockchain-enabled IoT and Fog-Cloud infrastructure for end users and transport data is challenging and needs to be solved in state-of-the-art studies.

### 1.2 Problem statement and research contributions

To answer all research questions, we solve this paper's scheduling research problem for transport data in fog cloud networks. In our case, we have different computing nodes, such as IoT, fog, and cloud. Due to the heterogeneity of the computing process, we designed the framework based on blockchain technology. We suggest the blockchain scheme to ensure the data security of IoT applications among different computing nodes. The federated learning scheme suggested the main goal is to train and process the data on different nodes and share it with the aggregated node for the final decision. This work introduces the MORFLB algorithmic schemes to minimize processing and transfer delays while maximizing long-term rewards. We focus on identifying known and unknown attacks on remote sensing data for transport (e.g., vehicle) applications. MORFLB incorporates multi-agent policies, proof-of-work hashing validation, and decentralized deep neural network training to achieve this objective with minimal delays. The infrastructure covers vehicle applications, decentralized fog, and cloud nodes, leveraging blockchain and reinforcement-federated learning techniques to enhance rewards through trial and error. Our study formulates a combinatorial problem that addresses various factors relevant to vehicle applications, making significant contributions to the field.

• This paper presents the smart-city-enabled IoT infrastructure platform for transport applications. We present the multi-objective reinforcement federated learning blockchain (MORFLB) framework. The aim is to minimize processing and transfer delays and maximize the long-term reward by identifying known and unknown attacks on remote sensing data for vehicle applications. MORFLB consists of multi-agent policies, proof-of-work hashing validation, and decentralized deep neural network training with minimum processing and transfer delays. MORFLB consists of vehicle applications, decentralized fog, and cloud nodes based on Blockchain reinforcement federated learning to improve the reward based on trial and error. The study formulates the combinatorial problem where the objectives are minima and maxima for vehicle applications.
• The study designs the reinforcement federated learning scheme, which tests and trains the model at different fog nodes and cloud nodes based on a deep neural network to identify the known and unknown attacks in the network.
• The study devises the blockchain scheme to process valid and immutable transactions among networks.
• The study presents adaptive scheduling, which maintains the quality of services of applications (e.g., deadline, delays, security) in the network.
• The study presents the simulator that is designed based on a mathematical model of the study and achieves optimal results compared to the existing simulator in practice.

The paper is organized as follows. Section 2 reviews related work, highlighting existing studies and their efforts in securing sensing data for vehicle applications. Section 3 describes the proposed system and problem formulation. Section 4 provides detailed steps of the proposed algorithms employed in this work. Section 5 discusses the simulation setup and presents the results and discussions. Finally, Section 6 concludes the study and outlines future directions for research.

## 2 Related work

These days, at the smart cities level, developed technologies such as Industry 4.0 contributed efficient inputs to transport data and applications. The IoT-based transport applications include mobile ticketing, transport location tracing, timetable of transports, fare compering, ticket validation, traffic prediction, and many more. However, these IoT-based applications offload data to the external for processing. Therefore, security is the key challenge at the smart level infrastructure for the transport data. We assume that the transport data consisted of end users and vehicle data collected from different servers in the smart cities. Furthermore, security and cybernetics issues related to sensing data in-vehicle applications within smart cities pose significant challenges. Existing literature suggests three machine learning approaches to address security, privacy, and attack identification: heuristic-based, program-based, and signature or pattern-based techniques. Signature-based cybersecurity and privacy methods have been proposed in previous studies to address these issues [1], [2], [3], [4]. This work analyzes and classifies three specific security and privacy issues: pattern recognition, object identification, and related resource consumption attacks on the network. However, the proposed strategies in these works consume more resources and determine the static network level security for the transport data.

To determine the dynamic analysis of the security on the network level for the transport data, the supervised and unsupervised machine learning techniques are presented. For instance, artificial neural networks (ANN), random forests, and support vector machines (SVM) have been suggested for self-autonomous vehicles [5], [6], [7], [8]. Fog and cloud services are deployed on the roadside and managed by the software-defined network (control and data planes). Convolutional neural networks (CNN) are employed to preprocess and train these models based on deep learning. These centralized fog and cloud networks utilize machine learning models to analyze security issues, such as denial of service attacks, phishing, and SQL injection intrusions during sensing data offloading. However, these studies only focused on single objectives, such as security for the transport data.

Machine learning and artificial intelligence techniques have been proposed for runtime and dynamic malware and intrusion detection in self-autonomous vehicle applications. The SARSA lambda framework with reinforcement learning has been introduced to identify malware patterns based on the network's different states, actions, rewards, and discount factors [9]. Deep Q-learning methods, including Deep Deterministic Policy Gradient and Asynchronous Advantage Actor-Critic Algorithm, have been suggested to detect Internet Controlling Messaging Protocol version-6 malware and intrusion cyber attacks at runtime [10], [11], [12], [13], [14], [15], [16], [17]. The main limitation of these proposed methods is that they are consuming due to the many rounds and states incurred with the longer delays in fog cloud networks.

Decentralized blockchain and federated learning solutions have been proposed in previous studies [18], [19], [20], [21], [22], [23]. Various blockchain frameworks, such as Ethereum, Corda, IBM, Fabric, and Sawtooth, have been explored, implementing private and public rights and authorization. Combined with blockchain technology, Federated learning allows the training and testing of models at different local nodes, which are then shared with the aggregated global node in the network. These studies have implemented centralized federated learning using blockchain technology to train and test cybernetics models for real-time sensing data in smart city vehicle applications [5], [24], [25], [26], [27]. Industry 4.0 enabled intelligent transport infrastructure for transport data content awareness based on blockchain considered in these studies [28], [29], [30], [31], [32], [33], [34]. The Industry 4.0 version enabled mobility, and ubiquitous Cargo transport enabled the processing of content-aware data and services. The goal was to process Cargo data based on valid hashing among communication and computing nodes. These studies considered the security and data flow constraints of transport applications. Therefore, delay, resource consumption runtime reward, and scheduling were not considered by these studies.

To the best of our knowledge, existing blockchain technologies have played a significant role in enhancing the data validity of public transport data across different computing nodes. However, these technologies have been plagued by delays, costs, and resource consumption when dealing with public transport data. Many machine learning and federated learning algorithms have been implemented with blockchain technologies. Nevertheless, the challenge of minimizing processing and transfer delays while maximizing long-term rewards by identifying known and unknown attacks on remote sensing data for vehicle applications has yet to be adequately addressed. Therefore, our work aims to address these new aspects related to public transport data.

## 3 Problem description and proposed system

**Figure 2:**
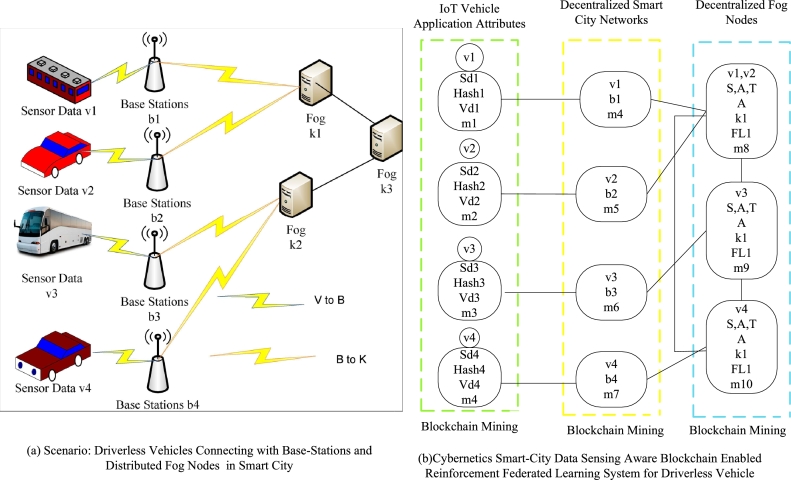
Multi-objectives reinforcement federated learning blockchain enabled Internet of things and Fog-Cloud infrastructure for transport data.

In the proposed system, we collect data from different vehicles and invoke services from wireless, fog, and cloud services. Therefore, data security and time-efficient systems only meet the requirements of these transport applications in the mobility environment. Therefore, we present the blockchain scheme to make the data transaction in a valid way on the heterogeneous nodes. The main advantage of our blockchain scheme is that we add only trusted nodes in the networks where the proposed blockchain scheme can process the valid data among different nodes. In our case, transport data of different vehicles is processed on different computing nodes, which means we divided the processes on different nodes. Therefore, we presented the federated learning-enabled scheme to process IoT transport data on different nodes based on the networks' given security, deadline, and time constraints. This study formulates a cybernetics smart-city data sensing-aware, blockchain-enabled reinforcement federated learning system for vehicles based on the Markov decision process environment with dynamic programming. The problem involves multiple layers and states with different attributes. The study devises a deep reinforcement policy and value schemes for scheduling with a reward constraint. The problem is combinatorial, with two main functions being optimized: convex and concave. The convex function aims to reduce communication and computation delays of vehicle applications, while the concave function seeks to maximize the cumulative reward of the vehicle applications. Cybernetics attacks are crucial in compromising the smart city's vehicle sensing data, which relies on blockchain technologies in distributed fog nodes. All fog nodes and sensors implement blockchain, and intrusion and attacks are trained and tested using the decentralized, federated learning paradigm, specifically deep neural networks. The study presents the cybernetics smart-city data sensing-aware blockchain-enabled reinforcement federated learning system for transport vehicles, as depicted in Fig. 2.

The system, depicted in Fig. 2 (a), represents all the Internet of Things (IoT) vehicles that generate sensing data through different sensors to the fog nodes. All the vehicles are connected to distributed base stations connected to the distributed fog nodes. The base stations are homogeneous, while the fog nodes are heterogeneous within the network. Fig. 2 (b) illustrates the proposed system, which comprises three layers: the IoT application layer, the network layer, and the fog node layer. Blockchain schemes, such as proof of work and hashing, are implemented across all layers to facilitate the distribution of valid data between base stations and fog nodes for processing. The problem formulation will be addressed in the subsequent subsection. Table 1 presents the notation used in the mathematical model for the problem formulation in this study.

**Table 1:** Problem symbolic formulation.

Symbol	Definition
*C*	Number of heterogeneous vehicles
*c*	Particular vehicle
*ϵ*_*c*_	Resource capability of vehicle
*V*	Number of Sensing Data Applications
*v*	The particular vehicular application of *V*
*sd*	It illustrates the sensing data
*sd*_*v*1_	Sensing data of IoT application *v*1
*vd*	Deadline of application *v*
*Hash*	Blockchain hashing of data
*Hash* ← *sd* ← *v*1	hash data of sensing data of application *v*1
*M*	Number of blockchain mining blocks
*m*	Particular mining of blocks
*B*	Number of homogeneous base stations
*b*	Particular base-station
*ϵ*_*b*_	Resource capability of b
*K*	Number of heterogeneous fog nodes
*k*	Particular fog node
*ζ*_*k*_	Speed of fog node
*ϵ*_*k*_	Resource capability of k
*FL*	Number of federating learning models
*fl*	Particular training and testing model
*Z*[*kw* ∈ *KW*]	Number of known attacks
*X*[*uw* ∈ *UW*]	Number of unknown attacks
$T_{sd}^{c}$	Execution delay data on vehicle
$T_{sd}^{b}$	Execution delay data on base-station
$T_{sd}^{k}$	Execution delay data on fog node
$E_{sd}^{c↔b}$	Round trip delay *c* and *b*
$E_{sd}^{b↔k}$	Round trip delay *b* and *k*
$E_{sd}^{k↔K}$	Round trip delay *k* and *K*
*S*	Number of states in reinforcement learning
*A*	Number of actions in reinforcement
*T*	Number of transition of states
*R*	Number of rewards of learning

### 3.1 Problem formulation

In the cybernetics enabled system, the study has *C* number of heterogenous vehicles, e.g., {c=1,…,C}, where each vehicle has processing capability $\epsilon_{c}$. The study assumes that, all vehicles can exploit the different number of applications, e.g., {v=1,…,V}. Each application *v* has the sensing data $v_{sd}$, and it has deadline $v_{sd}$. The study implements the *K* number of heterogeneous computing nodes, e.g., {k=1,…,K}. Every fog node *k* has distinct speed and computing resource capability in the network. The study implements *B* number of distributed homogeneous routing efficient base-stations, e.g., {b=1,…,B} with the same capability and resources in the network. There are *M* mining blockchian features in the fog nodes network, e.g., {m=1,…,M}. The problem formulated based on Markov decision process that have different states, actions and transition time, e.g., {s,a,t,…,S,A,T} in the discrete environment. The study derives the binary assignment of the application sensing data in the following way.

(1) $$x_{sd_{v},c,k,b}=\left\{ \begin{array}{cc} x_{sd_{v},c}=1\;Else\;0\; & Vehicle-Assigned,\\ x_{sd_{v},b}=1\;Else\;0\; & BS-Assigned,\\ x_{sd_{v},k}=1\;Else\;0\; & Fog-Assigned. \end{array}\right.$$

In equation (1), the assignment $x_{sd_{v},c}=1$ denotes that the particular sensing data assigned or read to execute on the particular vehicle *c* with the status 1, otherwise it will be a 0 assignment. In equation (1), the assignment $x_{sd_{v},b}=1$ denotes that, the particular sensing data assigned or read to execute on the particular base-station (BS) *b* with the status 1, otherwise it will be a 0 assignment. In equation (1), the assignment $x_{sd_{v},k}=1$ denotes that the particular sensing data assigned or read to execute on the particular fog node *k* with the status “1,” otherwise it will be a “0” assignment. The sensing data is processed on the different nodes such as vehicles, base-stations (BS), and fog nodes in order to maintain the security and hash validation on each node. Therefore, the blockchain mining and execution time of the all sensing data on the vehicles is determined as follows.

(2) $$c_{v}^{e}=\sum_{v=1}^{V}\sum_{c=1}^{C}\sum_{m=1}^{M}\sum_{s,a,t=1}^{S,A,T}(\frac{s1,a1,t,sd_{v},hash,m1}{\zeta_{c}})+E_{sd}^{c↔b}(\frac{sd_{v}}{bw})+Z[kw\in KW]+X[uw\in UW]\times x_{sd_{v},c}=1.$$

Equation (2) determines the execution time and communication time of data hashing, training, and testing validation in particular mining at the decentralized vehicles for all sensing data in different states with state, action, and transition timestamp with known and unknown attacks.

(3) $$b_{v}^{e}=\sum_{v=1}^{V}\sum_{c=1}^{C}\sum_{m=1}^{M}\sum_{s,a,t=1}^{S,A,T}(\frac{s1,a1,t,sd_{v},hash,m2}{\zeta_{b}})+E_{sd}^{b↔k}(\frac{sd_{v}}{bw})+Z[kw\in KW]+X[uw\in UW]\times x_{sd_{v},b}=1.$$

Equation (3) determines the execution time and communication time of data hashing, training, and testing validation in particular mining at the decentralized base stations for all sensing data in different states with state, action, and transition timestamp with known and unknown attacks.

(4) $$f_{v}^{e}=\sum_{v=1}^{V}\sum_{c=1}^{C}\sum_{m=1}^{M}\sum_{s,a,t=1}^{S,A,T}(\frac{s1,a1,t,sd_{v},hash,m2}{\zeta_{k}})+E_{sd}^{k↔K}(\frac{sd_{v}}{bw})+Z[kw\in KW]+X[uw\in UW]\times x_{sd_{v},k}=1.$$

Equation (4) determines the execution time and communication time of data hashing, training, and testing validation in particular mining at the decentralized fog nodes for all sensing data in different states with state, action, and transition timestamp with known and unknown attacks. The study predicts the reward for all sensing data applications in the following way on the different nodes.

(5) $$R++=c_{v}^{e}\leq vd+1+b_{v}^{e}\leq vd+1+f_{v}^{e}\leq vd+1.$$

Equation (5) determines the positive reward of sensing data under their deadline.

(6) $$R--=c_{v}^{e}\geq vd-1+b_{v}^{e}\geq vd-1+f_{v}^{e}\geq vd-1.$$

Equation (6) determines the negative reward of sensing data under their deadline. The commutative reward of the sensing data of vehicles determined in equation (7).

(7) CR=(R++)+(R−−),∀v,…,V.

Total delay of all the vehicles determined in equation (8).

(8) $$Total-Delay=c_{v}^{e}+b_{v}^{e}+k_{v}^{e},\;\forall v,\ldots ,V.$$

The formulation of the problem based on mathematical constraints determined in the following way. This study exactly has two objective functions such as convex function and concave function. The Total−Delay is the convex function and it has convex set such as, deadline *vd*, resources $\epsilon_{c,b,k}$, and training and testing *fl*, and known attacks Z[] and unknown attacks X[] must be satisfied. The study formulates the convex function in the following way.(9)
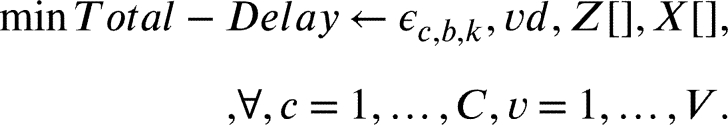
 Equation (9) is the convex optimization function with a convex set in terms of constraints that must be satisfied in the problem solution. The convex optimization problem is an NP-hard problem and must be executed under the polynomial time in the problem space for the vehicle applications. The study optimizes the concave function where commutative rewards of applications are increased during the processing in the system as determined in equation (10).

(10) max⁡CR←s,a,t,r,∀,c=1,…,C,v=1,…,V.

## 4 Proposed MORFLB algorithm methodology

The study proposes the Multi-Objective Reinforcement Federated Learning Blockchain (MORFLB) methodology to address convex and concave optimization problems that can be solved in polynomial time. The MORFLB incorporates various schemes, which are illustrated in Algorithm 1. Algorithm 1 provides a distinct approach to problem-solving, as initially defined in the work. Following the division of the problem into various parts, the study proceeds to design and train models using federated learning. Additionally, the study applies mining blockchain hashing processing to the data as it transitions between states within the network. Furthermore, the study devises a scheduler that efficiently schedules all workloads to minimize the total delay and maximize cumulative rewards in the network.

**Algorithm 1:**
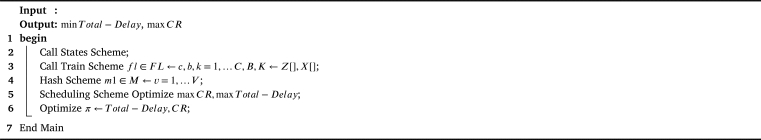
MORFLB.

### 4.1 Distributed reinforcement states

**Figure 3:**
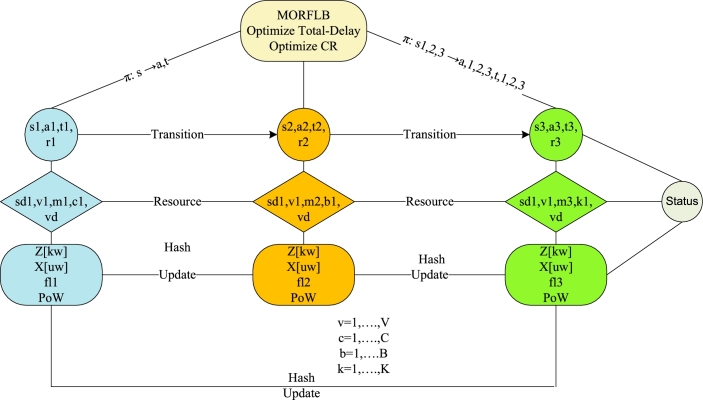
MORFLB framework cybernetics and federated learning efficient vehicle application data execution in networks.

The study formulates the MORFLB framework to optimize total delays and cumulative rewards (CR). The algorithmic process is illustrated in Fig. 3. Within the framework, the study divides the three-layer paradigms into distinct states, such as local vehicles, base stations, and fog nodes. Moreover, the study designs a policy wherein all states collectively optimize overall delays and total rewards for vehicle applications. Fig. 3 illustrates the comprehensive mechanism of the proposed policy scheme within the network. Each vehicle, base station, and fog node possesses distinct attributes related to vehicle workload processing, such as state (*s*1), action (*a*1), timestamp (*t*1), and reward (*r*1). Additionally, these nodes have attributes including vehicle identification (*v*1), hashing (*m*1), validation (*c*1), resource updates (*vd*), energy consumption (Z[kw]), execution time (X[uw]), and proof of work (PoW). Each node is converted into a hash using the SHA-256 algorithm and is subjected to training and testing using a deep neural network to detect known and unknown attacks denoted as *Z* and *X*, respectively. The study implements federated learning, meaning that decentralized blockchain hashing, based on SHA-256, is transmitted to another node, such as a base station, for training and validation within the network. The transfer of data from a vehicle to a base station is referred to as transition data, and if executed successfully without any violation of deadlines or attacks, it is rewarded. The study designs a deep neural network that trains and tests the proof of work method to validate the hashed data based on its signature and pattern during node transitions. The optimization objectives in the study are twofold: minimizing total delay and maximizing cumulative rewards. Successful execution of data with a status of “success” indicates that all workloads have been executed successfully across the network.

### 4.2 Federated learning enabled blockchain schemes for IoT transport

The study introduces a secure federated learning and blockchain-enabled policy where data undergoes processing on various computing nodes. Initially, employing federated learning at the local vehicle level, the proof of work method is utilized to train and test for cybernetic attacks within the network. Each transport IoT data is subjected to encryption and decryption based on the SHA-256 asymmetric mechanism within the distributed blockchain network. The workload of each vehicle application is transformed into a hash in the initial state, along with associated actions and timestamps. A reward system, encompassing positive and negative outcomes, is established for successful data execution without attacks on the vehicle node. A positive reward is granted when data is successfully offloaded to the base stations without encountering any attacks, enhancing cumulative rewards. Conversely, in the presence of an attack, the reward becomes negative, as evidenced. The primary objective centers on optimizing the policy denoted as *π* to minimize total delay and maximize rewards in the initial state. The resource and attack list is continuously updated to accommodate known and unknown attacks within the network. Should an attack occur, the system employs a deep neural network to detect it from the known list; if the attack is previously unidentified, it is promptly included in the list, with data stored in hash form at any node. Each base station receives hashed data from the vehicle during the transitional phase and converts it into a hash in the secondary state, recording relevant actions and timestamps. The reward system, mirroring that of the vehicle node, bestows positive rewards for successful, attack-free data execution, thus amplifying cumulative rewards. Conversely, in the event of an attack, the reward turns negative, following the pattern. The core objective remains optimizing the policy (*π*) to minimize total delay while maximizing rewards in the initial state. The resource and attack list remain subject to ongoing updates, accommodating known and unknown network attacks. In case of an attack occurrence, the system employs a deep neural network to detect it from the known list; if unidentified, it is promptly added to the list, and the data is maintained in hash form at any node. Finally, each fog node receives hashed data from the base station during the transitional phase, converting it into a hash in the tertiary state recording associated actions and timestamps. A reward system, analogous to the earlier nodes, operates by granting positive rewards for successful data execution without encountering attacks, thereby enhancing cumulative rewards. Conversely, in the presence of an attack, the reward becomes negative. The central objective remains optimizing the policy (*π*) to minimize total delay while maximizing rewards in the initial state. The resource and attack list persist in undergoing updates, addressing known and unknown network attacks. In the event of an attack, the system employs a deep neural network to detect it from the known list; if unidentifiable, it is promptly added to the list, with data maintained in hash form at any node.

The study devises the cybernetics federated learning and blockchain-enabled policy as shown in Algorithm 2 as the following steps.

• Initially, at the local vehicle, based on federated learning, the proof of work method train and test for the cybernetics attacks in the network. Each vehicle workload is encrypted and decrypted based on the SHA-256 asymmetric mechanism in the distributed blockchain network.
• Each vehicle application workload converts into a hash in the first state, actions, and timestamp. There is a reward (positive and negative) when data is executed successfully without any attacks in the vehicle node. When the data is successfully offloaded to the base stations without any attack, the reward will be positive and improve the commutative rewards, and if the attack exists, the reward will become negative, as shown from step-1 to step-13. The main objective is to optimize the policy, e.g., *π*, with the minimum total delay and maximize the reward in the first state. The resource and attack list is updated with known and unknown attacks in the network. If the attack happens, the system will detect it from the known list based on a deep neural network; if it is found unknown, it is included in the list, and the data is in the hashing form at any node.
• Each base station receives the hash data from the vehicle in the transition state and converts it into a hash in the second state, actions, and timestamp. There is a reward (positive and negative) when data is executed successfully without any attacks in the vehicle node. When the data is successfully offloaded to the base stations without any attack, the reward will be positive and improve the commutative rewards, and if the attack exists, the reward will become negative, as shown from step-1 to step-13. The main objective is to optimize the policy, e.g., *π*, with the minimum total delay and maximize the reward in the first state. The resource and attack list is updated with known and unknown attacks in the network. If the attack happens, the system will detect it from the known list based on a deep neural network; if it is found unknown, it is included in the list, and the data is in the hashing form at any node.
• Each fog node receives the hash data from the base station in the transition state and converts it into a hash in the third state, actions, and timestamp. There is a reward (positive and negative) when data is executed successfully without any attacks in the vehicle node. When the data is successfully offloaded to the base stations without any attack, the reward will be positive and improve the commutative rewards, and if the attack exists, the reward will become negative, as shown in steps 1 to 13. The main objective is to optimize the policy, e.g., *π*, with the minimum total delay and maximize the reward in the first state. The resource and attack list is updated with known and unknown attacks in the network. If the attack happens, the system will detect it from the known list based on a deep neural network, if it is found unknown, it is included in the list and the data in the hashing form at any node.

**Algorithm 2:**
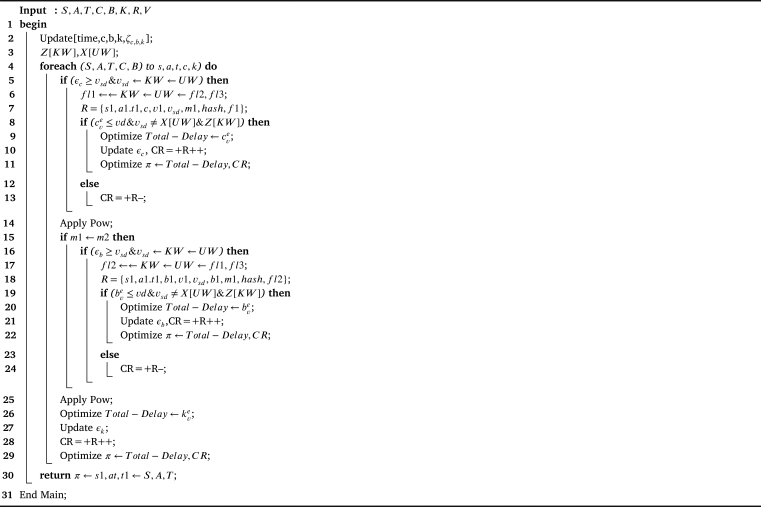
Blockchain enabled reinforcement federated learning system scheme.

## 5 Performance evaluation

In this phase, the study implemented various schemes on vehicle applications and computing nodes within the simulation environment. The simulation metrics and parameters utilized in the study are presented in Table 2. All the notations of the mathematical model are implemented in the configuration file as shown in Table 2. The study conducted training and testing of the cybernetics model using federated learning-enabled deep neural networks across various nodes, including vehicles, base stations, and fog nodes, to ensure data security and prevent attacks within the network. Table 3 showcases the training and testing models for both known and unknown attacks, which were deployed in the simulation tool to evaluate the performance of the method for vehicle applications in the network. Furthermore, the study developed a secure blockchain and reinforcement federated schemes-enabled infrastructure platform specifically designed for transport applications in distributed smart cities. The study introduces a secure smart city-enabled IoT infrastructure platform for transport applications, incorporating multiple services and ensuring system-wide security and data storage security.

**Table 2:** Experiments files and parameters.

Elements	Values
*V*	5 number of vehicle Applications
*v*1	Mass airflow sensor
*v*2	Engine Speed Sensor
*v*3	Real-Time Sensor
*v*4	Traffic Analyzing sensor
*v*5	Signals and Location Mapping
*C*	Number of vehicles
*c*1	Bus:100 GB ROM, 4 GB RAM
*c*2	Car:50 GB ROM, 4 GB RAM
*c*3	Metro:500 GB ROM, 16 GB RAM
*c*4	Mini-Car:10 GB ROM, 4 GB RAM
*B*	Number of base-stations
*b*1	50 GB ROM, 16 GB RAM
*b*2	50 GB ROM, 16 GB RAM
*b*3	50 GB ROM, 16 GB RAM
*b*4	50 GB ROM, 16 GB RAM
*b*5	50 GB ROM, 16 GB RAM
*K*	Number of three fog nodes
*k*1	500 GB ROM, 24 GB RAM
*k*2	1000 GB RAM, 32 GB RAM
*k*3	1500 GB RAM, 40 GB RAM
*S*,*A*,*T*	Number of states, actions and transition
*s*1	vehicle nodes
*a*1	Traffic and hashing execution
*t*1	Initial Transition

**Table 3:** Number of cybernetics attacks.

Attacks	Types	Methods	Status
*kw*_1_	Malware	Heuristic	Known
*kw*_2_	Viruses	Behavioral	Known
*kw*_3_	Worms	Signature	Known
*kw*_4_	Keyloggers	Signature	Known
*kw*_5_	Logic Bombs	Heuristic	Known
*kw*_6_	Spyware	Heuristic	Known
*uw*_1_	Malware	Deep Neural Pattern	Unknown
*uw*_2_	Viruses	Deep Neural Pattern	Unknown
*uw*_3_	Worms-fire	Deep Neural Pattern	Unknown
*uw*_4_	Trojan	Deep Neural Pattern	Unknown
*uw*_5_	Toolkits	Deep Neural Pattern	Unknown
*uw*_6_	Fileless	Deep Neural Pattern	Unknown

### 5.1 Smart-city data and number vehicle and services scenario and implementation

**Figure 4:**
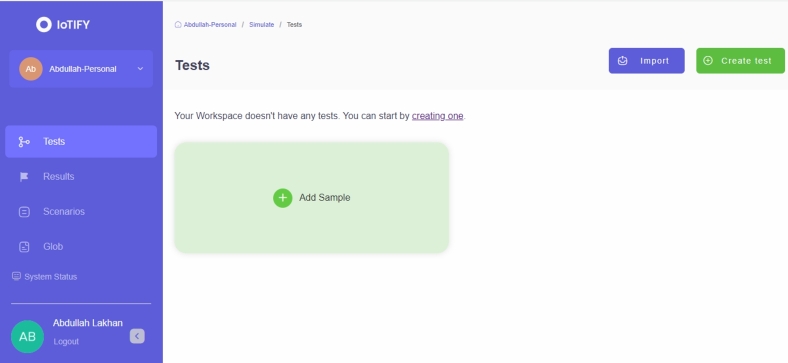
Smart-city NETSIM smart-city simulator for transport applications.

**Figure 5:**
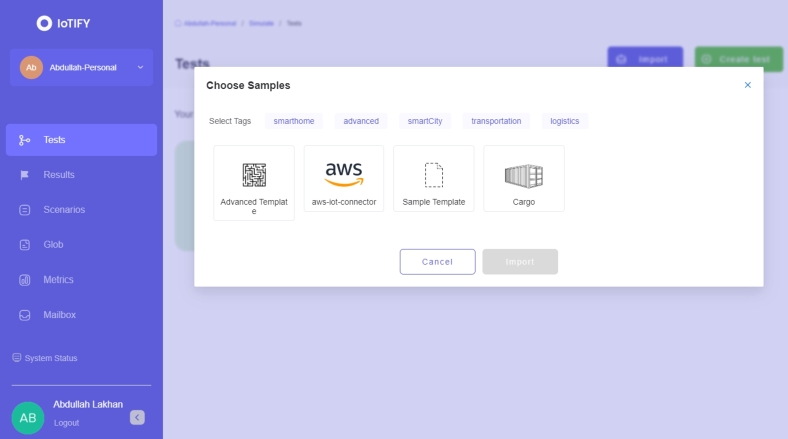
Infrastructure including base-stations and fog and cloud nodes.

The study developed the Transport NetSim simulator as shown in Fig. 4 based on transport applications in smart cities. The simulator incorporates various smart city services within a fog and cloud-based distributed infrastructure for transport applications. It encompasses design models, algorithms, city representations, data, and blockchain models. Through the simulation, the study demonstrates that service availability and delays vary during different peak hours. For instance, during peak hours, such as 9 a.m. to 12 p.m., higher delays are observed compared to lighter hours within the simulation environment for transport applications in smart cities. Fig. 4 and Fig. 5 show the initial level of software netsim based on existing IoTfy netsim services [35] for the experiment.

**Figure 6:**
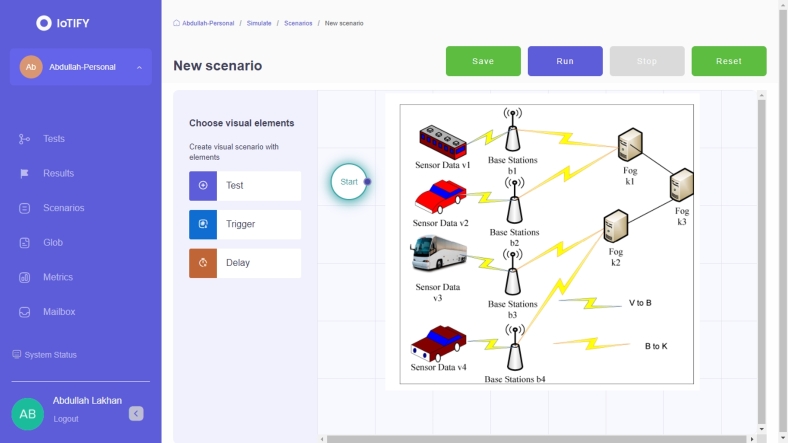
IoT transport applications implemented in infrastructure including base-stations and fog and cloud nodes.

**Figure 7:**
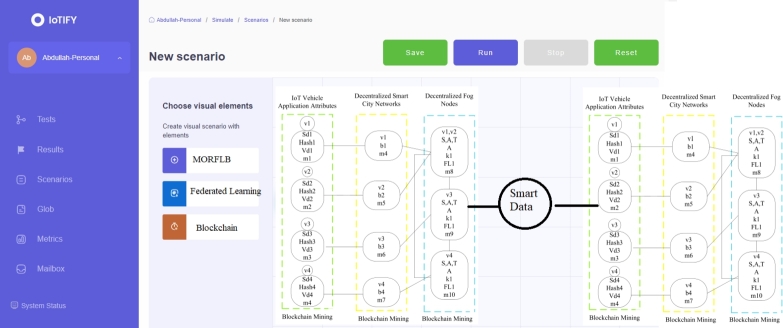
Blockchain-Fog-Cloud-federated-learning-MORFLB.

Fig. 6 shows the implementation of transport applications along with the base stations and fog and cloud nodes with both communication models, such as IoT vehicle to fog node and fog node to Cloud for the smart city services execution in the system. The designed system demonstrates the service delays of the nodes in different time zones in the smart city for the IoT transport applications in the system as shown in Fig. 7. We implemented blockchain methodology, proof of validation, MORFLB algorithms schemes, and federated learning for transport applications based on fog, cloud, and base stations. We implemented the MORFLB based on open application programming API and transport data.

### 5.2 Dataset description

We demonstrated the data processing in different processes as shown in Fig. 7. The public transport data file encompasses a variety of transport data types, including text and numeric data for local cars, metros, trains, and buses. For experimental purposes, we have designed the distribution of public transport data at the smart city level. The simulation incorporates 72,000 metro data, 80,000 bus data, 20,000 train data, and 25,000 car data, along with their corresponding schedules, timetables, and routes. Numeric data, such as available time, route time, and starting and ending points of public transport, have also been integrated. This data was extracted from a CSV file accessible at the URL: ssb.no/en/transport-og-reiseliv/landtransport/statistikk/innenlandsk-transport. The simulation focuses on sharing public transport data among different public transport owners to enhance travel safety, planning, and overall quality of transport services for customers. The public transport data is collected from sensors like the Global Positioning System (GPS), surveillance signal cameras, and routing servers. This collected data includes information about the location and types of transportation, which is then analyzed to identify patterns and improve customer travel experiences. These data are aggregated and shared among various transport servers responsible for road and public transport activities. Given the sensitive nature of customer data, such as tickets, biodata, and other critical information, ensuring data security is of utmost importance in smart cities. We aim to efficiently distribute and share this data among different public nodes using blockchain technology to ensure data validation. The infrastructure spans a range of 500 kilometers, and diverse nodes with different characteristics communicate.

We implemented known and unknown data intrusions data in distributed communication based on blockchain technologies. We integrated the TCP/IP [36] communication protocols between vehicles and servers. We combined 42 qualitative and quantitative features on known and unknown blockchain technologies to determine their system performance. The data intrusion (e.g., known and unknown) attacks are analyzed based on the existing dataset and URL given in the data statements. However, we modified the dataset according to the parameters. There the modified dataset contains the blockchain transaction failure status, computing node execution status, source, destination, protocols, attack type, known attack name, unknown attack type, and many other features. The dataset is available on this URL: https://github.com/Abdullah-Lakhan/Intrusion-Dataset/tree/main.

### 5.3 Result discussion

**Figure 8:**
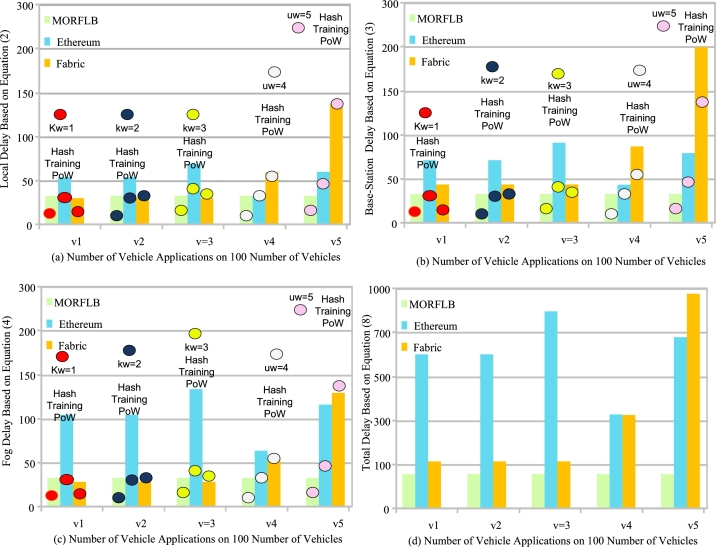
Total delay performance of blockchain frameworks to detect intrusions for vehicle applications.

The study incorporated baseline approaches that utilize deep reinforcement learning and blockchain technology for scheduling vehicle applications within distributed vehicle base-station fog networks. For instance, these baseline approaches include DISCOUNT (Dispatch of UAVs for Urban VANETs) [26], as well as DRL (Deep Reinforcement Learning) [20], [21], [22], and MHRL (Multi-Headed Reinforcement Learning) [27]. These approaches were implemented in the configuration file during the simulation. The simulation environment also incorporates the Ethereum blockchain and Fabric blockchain frameworks. Fig. 8 illustrates the performance comparison of all baseline methods and the proposed methods when running vehicle applications on vehicle nodes, base stations, and fog nodes, considering both known and unknown cybernetics attacks in the system. Known attacks are represented by {kw=1,…,Kw}, while unknown attacks are represented by {uw=1,…,Uw}. The performances of these attacks are displayed in Fig. 8.

We analyze the performance of individual nodes separately since processing nodes are decentralized and can detect and analyze data in a decentralized manner. Fig. 8 (a) showcases the local delay and the performance of both known and unknown cybernetics attacks. The proposed MORFLB approach outperforms existing blockchain frameworks (e.g., Ethereum and Fabric) in terms of processing delays and handling cybernetics attacks. The study designed a federated learning-enabled blockchain network along with a deep neural network to detect and process vehicle application data within the distributed network. In our case, the trained and tested models of all nodes are shared with each other, and blockchain validation based on hashing only identifies invalid transactions but does not detect cyber attacks in the system. Consequently, the performance of existing blockchain technologies degrades when there are more attacks than invalid hashes in the network. Existing blockchain technologies fail to detect runtime attacks, whether known or unknown. On the other hand, the decentralized federated learning blockchain trains and tests models at each node, enabling easy and efficient processing without significant delays, as demonstrated in all nodes in Fig. 8 (b), (c), (d). Fig. 8 (b) presents the performance of base stations data validation and intrusion detection for vehicle data. The results indicate that the proposed scheme improves processing delays and rewards compared to existing schemes. Fig. 8 (c) reveals that the system selects optimal fog nodes for processing, resulting in reduced processing delays and improved rewards during the execution of vehicle workloads in the network. Fig. 8 (d) illustrates that the proposed approach minimizes overall delays compared to all existing baseline approaches within the network.

**Figure 9:**
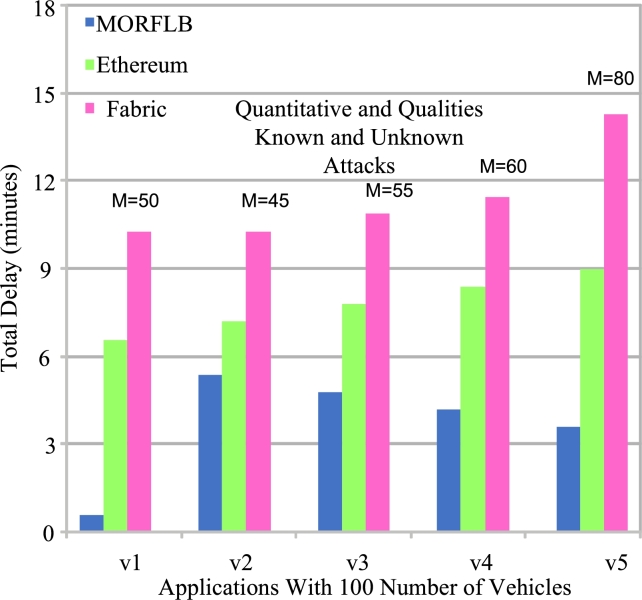
Performance of known and unknown attacks on blockchain blocks during processing of vehicle applications.

During the simulation, we conducted various known and unknown attacks on the blockchain, targeting different requests and blocks. The attacks were quantitatively and qualitatively determined, and the dataset consisted of various features such as transaction duration, offloading type protocols, available node services, status flags, blockchain blocks, and source and destination addresses, among others. Our primary objective was to prevent blockchain transactions from being attacked and incurring significant delays. Fig. 9 depicts the execution of vehicle applications on distributed fog cloud networks. Each vehicle application is associated with different blockchain blocks to process their workloads. For example, v1∼M=50, v2∼M=45, v3∼M=55, v1∼M=60, and v1∼M=80. The known and unknown attacks are characterized by qualitative and quantitative features, including numeric and text values. Fig. 9 illustrates the performance of blockchain technologies for different applications with varying levels of delays. For instance, for application *v*1, the MORFLB blockchain incurs approximately 2 minutes of delay. The main reason for this improvement is that our blockchain scheme was trained on valid transactions, hashing, and validation schemes. On the other hand, Ethereum and Fabric blockchain still suffer from long delays. This can be attributed to these blockchain frameworks not allowing invalid transactions, resulting in delays and missed application deadlines.

**Figure 10:**
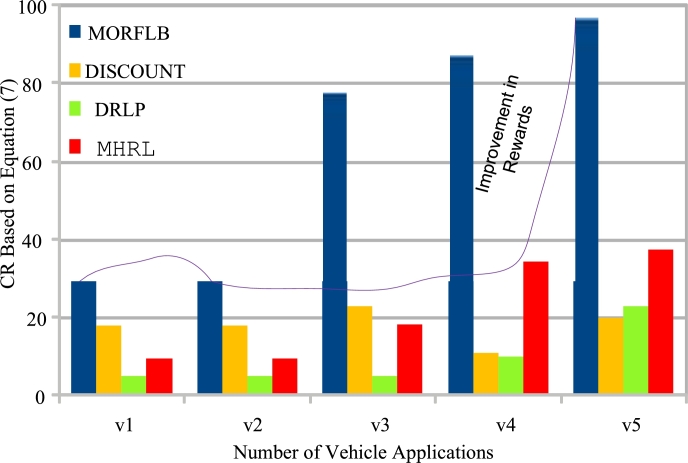
Commutative rewards of vehicle applications of different schemes.

Fig. 10 demonstrates that the proposed approach achieves optimal rewards and consistently improves upon the rewards compared to existing studies. This improvement can be attributed to existing blockchain technologies only validating hashes using proof-of-work methods and rejecting invalid transactions among different nodes. However, when subjected to different attacks, these existing blockchain frameworks' detection and removal times increase. Therefore, Fig. 10 highlights the effectiveness of decentralized, federated learning with blockchain technology in achieving improved and valid results, even in the presence of both known and unknown attacks within vehicle networks. The study focuses on increasing cumulative rewards across different vehicle nodes, base stations, and fog nodes while preventing resource leakage at each node during the processing of applications, as depicted in Fig. 10.

### 5.4 Finding and limitation of MORFLB

We devised the multi-objective reinforcement federated learning blockchain (MORFLB) framework to process the secure, delay, and deadline-efficient data on different computing nodes. The main limitation of the existing blockchain technologies is that they have played a significant role in enhancing the data validity of public transport data across different computing nodes. However, these technologies have been plagued by delays, costs, and resource consumption when dealing with public transport data. These algorithms, e.g., DISCOUN, DRLP, MHRL, Ethereum, and Fabric, have been implemented with blockchain technologies. None of the methods minimizes computation processing and transfer delays while maximizing long-term rewards by identifying known and unknown attacks on remote sensing data for transport data except MORFLB. The main advantages of MORFLB are to optimize cost, delay, resource consumption, meet deadlines of tasks, and security as compared to existing studies for transport data. However, due to large and distributed computing nodes, many challenges exist in MORFLB. The initial challenge is fault-tolerant, where many tasks could fail due to transient unavailability of resources. Another reason is that if the cyber-attacks occur on the nodes, the failure is due to an attack on the nodes. It leads to failure and violation of all tasks deadlines in the computing nodes. Another limitation is that services boot-up time is larger in our MORFLB computing nodes; therefore, if a failure task is scheduled to another node, it is a resource of the node with boot-up delay and consumes much more time and resources of the computing nodes. In future work, we will add more constraints in MORFLB, such as heterogeneous computing time, hybrid cost, resource scalability, and data modality. Resolving these factors is pivotal for managing extensive data and furnishing streamlined services for public transportation data while acknowledging the related security and financial limits. Energy efficiency, data filtering, and processing dimensions still need to be addressed in the present MORFLB iteration. Subsequent work will encompass these limitations and confront these matters when grappling with public transport data.

## 6 Conclusion and future work

This study formulated a secure smart-city data sensing-aware, blockchain-enabled reinforcement federated learning system for vehicles based on the Markov decision process environment with dynamic programming. The problem involves multiple layers and states with different attributes. The study devises a deep reinforcement policy and value schemes for scheduling with a reward constraint. The problem is combinatorial, with two main functions being optimized: convex and concave. The convex function goal in our work was to reduce communication and computation delays of vehicle applications. In contrast, the concave function seeks to maximize the cumulative reward of the vehicle applications. Cybernetics attacks are crucial in compromising the smart city's IoT transport data, which relies on blockchain technologies in distributed fog and cloud computation machines. In the result discussed, we showed the MORFLB obtained optimal results compared to existing transport data methods. MORFLB approach incorporates multi-agent policies, proof-of-work hashing validation, and decentralized deep neural network training, resulting in minimal processing and transfer delays. By utilizing Blockchain reinforcement federated learning, MORFLB optimizes rewards through trial and error, encompassing vehicle applications, decentralized fog, and cloud nodes. The study addresses a combinatorial problem with multiple objectives for vehicle applications, aiming to minimize certain factors and maximize others. The experimental results highlight the effectiveness of MORFLB in reducing processing and transfer delays while maximizing rewards compared to existing approaches.

However, MORFLB does have certain research limitations that will be addressed in future work. The framework needs to consider processing, resource, and data modality costs. Addressing these aspects is crucial for dealing with big data and providing efficient services for public transport data, considering the associated security and cost constraints. Furthermore, energy efficiency, data filtration, and processing aspects have yet to be considered in the current version of MORFLB. Future work will incorporate these constraints and address these issues when handling public transport data.

## Funding

This article was co-funded by the 10.13039/501100000780European Union under the REFRESH-Research Excellence For REgion Sustainability and High-tech Industries project number CZ.10.03.01/00/22_003/0000048 via the Operational Programme Just Transition. Also, this work was supported by 10.13039/501100001823The Ministry of Education, Youth and Sports of the Chezk Republic conducted by VSB-Technical University of Ostrava, Czechia under Grants SP2023/039 and SP2023/042.

## Ethical statements

There is no ethical issues in the manuscript, the data, figures, and results are original and not published in any other places.

## CRediT authorship contribution statement

**Mazin Abed Mohammed:** Conceptualization, Formal analysis, Methodology, Writing – review & editing. **Abdullah Lakhan:** Conceptualization, Formal analysis, Methodology, Writing – review & editing. **Karrar Hameed Abdulkareem:** Conceptualization, Formal analysis, Methodology, Writing – review & editing. **Mohd Khanapi Abd Ghani:** Formal analysis, Investigation. **Haydar Abdulameer Marhoon:** Formal analysis, Investigation. **Jan Nedoma:** Funding acquisition, Project administration. **Radek Martinek:** Funding acquisition, Project administration.

## Declaration of Competing Interest

The authors declare that there is no conflict of interests.

## Data Availability

We implemented the available physical data of public transport, which is available on the following URL. The data is available in CSV form. We implemented the offloading and scheduling and performed the blockchain technology for execution: ssb.no/en/transport-og-reiseliv/landtransport/statistikk/innenlandsk-transport. The intrusion dataset (e.g., known and unknown) attacks are implemented, which is downloaded from this ordinal source: https://www.kaggle.com/datasets/sampadab17/network-intrusion-detection. However, we modified it with both qualitative and qualitative according to our parameters. The modified dataset is available here: https://github.com/Abdullah-Lakhan/Intrusion-Dataset/tree/main.
